# Identification of an Aptamer With Binding Specificity to Tumor-Homing Myeloid-Derived Suppressor Cells

**DOI:** 10.3389/fphar.2021.752934

**Published:** 2022-01-21

**Authors:** Shaohui Tian, Thomas Welte, Junhua Mai, Yongbin Liu, Maricela Ramirez, Haifa Shen

**Affiliations:** ^1^ Department of Nanomedicine, Houston Methodist Academic Institute, Houston, TX, United States; ^2^ Department of Gastrointestinal Surgery, The Third Xiangya Hospital of Central South University, Changsha, China; ^3^ Weill Cornell Medical College, White Plains, NY, United States

**Keywords:** tumor-targeted delivery, myeloid-derived suppressor cell, aptamer, structure-activity relationship, G-quadruplex

## Abstract

Myeloid-derived suppressor cells (MDSCs) play a critical role in tumor growth and metastasis. Since they constantly infiltrate into the tumor tissue, these cells are considered as an ideal carrier for tumor-targeted drug delivery. We recently identified a DNA-based thioaptamer (T1) with tumor accumulating activity, demonstrated its potential on tumor targeting and drug delivery. In the current study, we have carried out structure-activity relationship analysis to further optimize the aptamer. In the process, we have identified a sequence-modified aptamer (M1) that shows an enhanced binding affinity to MDSCs over the parental T1 aptamer. In addition, M1 can penetrate into the tumor tissue more effectively by hitchhiking on MDSCs. Taken together, we have identified a new reagent for enhanced tumor-targeted drug delivery.

## Introduction

Multiple physical and biological barriers block drug molecule penetration in the tumor tissue, rendering most therapeutic agents ineffective (Blanco et al., 2015; Rosenblum et al., 2018). Thus, there is a high demand for developing new drug formulations and identifying new delivery routes that facilitate tumor enrichment and intratumor penetration of anti-cancer therapies (Chen et al., 2017; Li et al., 2020). Cell-mediated drug delivery is one of such options (Xue et al., 2017; Kutova et al., 2019). With this promising approach, adequate cells can serve as a carrier to drive therapeutic agents deep into the tumor (Combes et al., 2020). Cell-based drug delivery is believed to possess a number of advantages over the conventional drug delivery approaches, such as active delivery with high selectivity, prolonged retention, and sustained drug molecule release (Su et al., 2015; Huang et al., 2018; Zhang et al., 2021). Various tumor-associated cell types can serve as the vehicle for cell-based drug delivery making the best use of their natural tendency on tumor homing in response to tumor-secreted chemoattractants (Tang et al., 2021). Indeed, many immune cells including T cells, monocytes and neutrophils, macrophages have all been tested as the vehicle for tumor-targeted drug delivery (Nakamizo et al., 2005; Huang et al., 2015; Xia et al., 2020; Ye et al., 2020; Qu et al., 2021).

Most studies on cell-mediated drug delivery have mainly focused on packaging carrier cells with a therapeutic cargo *ex vivo* (Timin et al., 2018; Tang et al., 2021). However, viability and migration property of the cells can be altered during the drug-loading process; in addition, cell manipulation is a costly and sophisticated process (Dekaban et al., 2013). In this regard, a direct *in situ* loading strategy provides a better alternative (Feng et al., 2020). However, in order to achieve a high targeting efficiency, it is essential to have a reagent with high binding affinity and specificity to the carrier cell that allows for effective drug internalization in circulation.

Myeloid-derived suppressor cells (MDSCs) are a heterogeneous population of immature myeloid cells that constitutes an important part of the immunosuppressive tumor microenvironment (Veglia et al., 2018). They play a critical role in tumor progression and metastasis (Tian et al., 2019; Swierczak and Pollard, 2020), and are correlated with poor prognosis (Zhang et al., 2017). It has been reported that a large number of MDSCs are recruited to the tumor tissue and pre-metastatic niches during tumor expansion (Bosiljcic et al., 2019; Hoffmann et al., 2019; Cassetta et al., 2020). Compared with other immune cells, most of which preferentially migrate to lymphoid organs or livers, MDSCs show more specific tumor tropism (Eisenstein et al., 2013). Both the abundance and high mobility make circulating MDSCs an ideal vehicle for transporting drug molecules or particles from bloodstream into the neoplastic lesion (Chandra and Gravekamp, 2013).

Aptamers are single-stranded oligonucleotides folded into unique three-dimensional structures. They can bind to both small and macro-molecules with high affinity and specificity (Zhang et al., 2013). In addition, aptamers offer a number of advantages over antibodies such as lower immunogenicity, less complexity, and easy to produce (Zhou and Rossi, 2017; Kratschmer and Levy, 2018). However, they also suffer from certain disadvantages such as low bioavailability and stability, and rapid clearance from the body (Sun and Zu, 2015; Odeh et al., 2019). Thus, there is a need to identify aptamers with desirable physical and chemical properties for drug delivery. In our previous work, we applied *in vivo* systematic evolution by exponential enrichment (SELEX) screening and identified a novel DNA thioaptamer (T1) that showed tumor tropism (Liu et al., 2018; Mai et al., 2018). Interestingly, the T1 aptamer could bind to both MDSCs and cancer cells, thus serving as an affinity moiety for tumor-targeted drug delivery. In addition, unlike other aptamers designed to interact with tumor-associated MDSCs (De La Fuente et al., 2020), the T1 aptamer obtained from our *in vivo* selection binds to both tumor-infiltrated MDSCs and tumor-homing MDSCs in circulation, which may contribute to blood retention and ultimately enhanced tumor accumulation. In the current study, we have taken an additional effort to perform structure-activity relationship analysis on T1 aptamer. During the process, we have unmasked key sequence and structural features that determine MDSC-binding activity from the aptamer. By incorporating these features, we have identified a new aptamer (M1) with enhanced MDSC-binding ability.

## Materials and Methods

### Oligonucleotides

All oligonucleotides used in this study were synthesized by Integrated DNA Technologies (IDT, United States). Sequences information for individual aptamers are provided in Table 1. The oligonucleotides were resuspended in water to a final concentration of 100 µM as the stock solution. Purity of each sample was examined with HPLC.

**TABLE 1:** Aptamer sequences with G4 Hunter scores and predicted quadruplexes.

Sequence	Quadruplexes Found	G4 Hunter Score
CGC​TCG​A*TA*GA*TCG​A*GCT​TCG​CTC​GA*TGT​GGT​GTT​GTG​GGG​GCT​TGT​A*TTG​GTC​GA*TCA*CGC​TCT​A*GA*GCA*CTG	1	1.2
A*T CCA GAG TGA CGC AGC A*CT A*CT GGA* CTT CA*T CGG A*GC TAG GTC A*TC GCT TGC A*TG CA*T GGA* CA*C GGT GGC TTA	0	n/a
CGC​TCG​A*TA*GA*TCG​A*GCT​TCG​CTC​GA*TGT​GGT​GTT​GTG​GGG​GCT​TGT​A*TTG​GTC​GA*TCA*CGC​TCT​A*GA*GCA*CTG	1	1.2
CGC​TCG​A*TA*GA*TCG​A*GCT​TCG​CTC​GA*TCA*CGC​TCT​A*GA*GCA*CTG	0	n/a
CGC​TCG​A*TA*GA*TCG​A*GCT​TCG​CTC​GA*TGT​GGT​GTT​GTG​GGG​GCT​TGT​A*TTG​GTC​GA*TCA*C	1	1.2
CGC​TCG​A*TA*GA*TCG​A*GCT​TCG​CTC​GA*TGT​GGT​GTT​GTG​GGG​GCT​TGT​A*TTG	0	n/a
CGC​TCG​A*TA*GA*TCG​A*GCT​TCG​A*TCG​A*TGT​GGT​GTT​GTG​GGG​GCT​TGT​A*TTG​GTC​GA*TCA*C	1	1.2
CGC​TCG​A*TA*GA*TCG​A*GCT​TCG​CTC​GA*TGT​GGT​GTT​GTC​TTG​TA*TTG​GTC​GA*TCA*C	0	n/a
CGC​TCG​A*TA*GA*TCG​A*GA*TTC​GCT​CGA*TGT​GGT​GTT​GTG​GGG​GCT​TGT​A*TTG​GTC​GA*TCA*C	1	1.2
CGC​TCG​A*TA*GTT​CTC​GA*GCT​TCG​CTC​GA*TGT​GGT​GTT​GTG​GGG​GCT​TGT​A*TTG​GTC​GA*TCA*C	1	1.2
CGC​TCG​A*TA*GA*TCG​A*GCT​TCG​A*TCG​A*TGT​GGT​GTT​GTC​TTG​TA*TTG​GTC​GA*TCA*C	0	n/a
CGC​TCG​A*TA*GA*TCG​A*GCT​TCG​CTC​GA*TGT​GGT​GTT​GTG​GGG​GCT​TGG​TCG​A*TCA*C	1	1
CGC​TCG​A*TA*GA*TCG​A*GCT​TCG​A*TCG​A*TGT​GGG​GTT​GTG​GGG​GCT​TGT​A*TTG​GTC​GA*TCA*C	1	1
CGC​TCG​A*TA*GA*TCG​A*GCT​TCG​A*TCG​A*TGT​GGG​GTT​GTG​GGG​GCG​GGT​A*TGG​GTC​GA*TCA*C	1	1.2
CGC​TCG​A*TA*GA*TCG​A*GCT​TCG​A*TCG​A*TGT​GGT​GTT​GGG​GGG​GCT​TGT​A*TTG​GTC​GA*TCA*C	1	0.8
CGC​TCG​A*TA*GA*TCG​A*GCT​TCG​A*TCG​A*TGT​GGT​GTT​GTG​GGA*GCT​TGT​A*TTG​GTC​GA*TCA*C	0	0
CGC​TCG​A*TA*GA*TCG​A*GCT​TCG​A*TCG​A*TGT​GGT​GTG​GGG​GTG​TCT​TGT​A*TTG​GTC​GA*TCA*C	1	1.2
CGC​TCG​A*TA*GA*TCG​A*GCT​TCG​A*TCG​A*TGT​GGT​GTT​GTC​TTG​TA*TGG​GGG​TGG​TCG​A*TCA*C	1	1.2
CGC​TCG​A*TA*GA*TCG​A*GCT​TCG​CGC​GA*TGT​GGT​GTT​GTG​GGG​GCT​TGT​A*TTG​GTC​GCT​CA*C	1	1.2
CGC​TCG​A*TA*GA*TCG​A*GCT​TCG​CTG​CGA*TGT​GGT​GTT​GTG​GGG​GCT​TGT​A*TTG​GTC​GCA*TCA*C	1	1.2
CGC​TCG​A*TA*GA*TCG​A*GCT​TCG​CGC​GA*TGT​GGT​GTT​GTG​GGG​GCT​TGT​A*TTG​GTC​GCG​GA*C	1	1.2
CGC​TCG​A*TA*GA*TCG​A*GCT​TCG​CTC​GCG​A*TGT​GGT​GTT​GTG​GGG​GCT​TGT​A*TTG​GTC​GCG​A*TCA*C	1	1.2
CGC​TCG​A*TA*GA*TCG​A*GCT​TCG​CTG​A*TCG​A*TGT​GGT​GTT​GTG​GGG​GCT​TGT​A*TTG​GTC​GA*TCA*TCA*C	1	1.2
CGC​TCG​A*TA*GA*TCG​A*GCT​TCG​A*TCG​A*TGT​GGT​GTT​GTG​GGG​GCT​TGT​A*TTG​GTC​GA*TCG​C	1	1.2
CGC​TCG​A*TA*GA*TCG​A*GCT​TCC​GA*TCG​A*TGT​GGT​GTT​GTG​GGG​GCT​TGT​A*TTG​GTC​GA*TCG​A*C	1	1.2

### Cell Culture

The human chronic myelogenous leukemia (CML) cell line K562 and human acute myeloid leukemia (AML) cell line Molm14 were cultured in RPMI 1640 (Corning, United States) supplemented with 10% fetal bovine serum (FBS, GIBCO, United States), 100 U/ml penicillin and 100 µg/ml streptomycin (Cellgro, Corning, United States) at 37°C with 5% CO_2_. Murine 4T1 mammary carcinoma cells were cultured in Dulbecco’s Modified Eagle’s Medium (DMEM, Corning, United States) supplemented with 10% FBS, 100 U/ml penicillin and 100 µg/ml streptomycin at 37°C with 5% CO_2_. Peripheral blood mononuclear cells (PBMCs) were collected from 4T1 tumor-bearing mice, lysed with an ACK lysis buffer (KD, United States) for 5 min on ice, and then maintained in complete DMEM with 55 µM 2-mercaptoethanol (Gibco, United States).

### Murine Tumor Model

All procedures in animal studies were carried out strictly following a protocol approved by the Institutional Animal Care (IACUC) at Houston Methodist Research Institute. Female Balb/c mice (6 to 8-week-old) were purchased from the Jackson Laboratory. 4T1 orthotopic breast cancer model was established by inoculating 5×10^5^ 4T1 cells in 100 μl phosphate buffered saline (PBS)/Matrigel (Corning, United States) in the left mammary fat pad.

### Detection of Free and Cell-Associated Aptamers in Circulation

For aptamer *in vivo* partition experiments, 4T1 tumor-bearing mice were treated intravenously (*i.v.*) by tail with 0.4 nmol Cy5-labeled aptamers (Cy5-aptamer) in 200 µl PBS. Periphery blood samples were collected 30 min and 4 h post-injection. They were processed with centrifugation, and cell pellets were treated with an ACK lysis buffer for 5 min on ice. After one round of wash, cells were resuspended in 100 μl 2% FBS, and fluorescent intensities from all samples were measured with a Biotek Synergy H4 Hybrid Reader, and further confirmed with flow cytometry.

### *In vitro* and *ex vivo* Aptamer Binding Assays

Cells were resuspended in a PBS-based binding buffer containing 2% FBS, 10% glucose, 5 mM MgCl_2_, 0.1 mg/ml salmon sperm DNA (ssDNA, R&D), and 100 µg/ml yeast tRNA (Invitrogen, United States) for 5 min on ice, following a previously described protocol with slight modification (Sefah et al., 2010). To measure cell binding by aptamers *in vitro*, 40 nM Cy5-aptamer was added to 1 million K562 or Molm14 cells. The cell suspension was maintained on ice for 30 min, and unbound aptamer was washed out before samples were applied for flow cytometry analysis. To examine aptamer binding to PBMCs *ex vivo*, 0.5 million PBMCs were mixed with 200 nM Cy5-aptamer in a 100 µl binding solution. Cells were washed with 2% FBS in PBS before they were applied for flow cytometry analysis.

### G4 Hunter Application

Sequences were uploaded to a web-based server named DNA analyser (http://bioinformatics.ibp.cz), and the system provided automated analysis on G-quadruplex motifs (Brázda et al., 2019). G4 Hunter parameters were set as recommended.

### Aptamer Separation With Agarose Gel Electrophoresis

Aptamers were separated with electrophoresis on both denatured and non-denatured agarose gels. To separate on a non-denatured gel, 5 µM sample in 10 µl PBS was loaded into each well in a 3% agarose gel prepared with GelRed (Biotium, United States) in tris-acetate-EDTA buffer. GeneRuler Low Range DNA Ladder (Thermo Scientific, United States) ranging from 25 bp to 700 bp was used as standard markers. To separate on a denatured gel, 5 µM sample in 10 µl PBS was heated at 70°C for 5 min, and then chilled on ice for 3 min before it was loaded onto a 2.5% agarose gel in an alkaline electrophoresis buffer containing 30 mM NaOH and 2 mM EDTA. Electrophoresis was run at a constant voltage of 90 V for 1.5 h.

### Flow Cytometry Analysis

To identify binding capability of each aptamer on the K562 and Molm14 cell lines, cells were resuspended in 2% FBS and stained with DAPI at a 1:10,000 dilution before they were applied for flow cytometry analysis. To assess aptamer binding on PBMCs, cells were first incubated with fluorescently labeled antibodies, and then stained with DAPI before flow cytometry analysis on an LSRII Flow Cytometer or a BD FACS Fortessa (Bronte et al., 2016). Antibodies used for flow cytometry analysis included FITC-CD45 (BD Biosciences, United States), APC-Cy7- CD45 (BD Biosciences, United States), PE-CD11b (Tonbo, United States), AF700-Ly6G (Biolegend, United States), FITC-Ly6G (Biolegend, United States), PE-Cy7-Ly6C (Biolegend, United States). Results were analyzed using the Flowjo software.

### Aptamer Penetration Into Tumor Spheroids

To generate 4T1 tumor spheroids, 3,000 4T1 cells were added into each well in an ultralow attachment round bottom microplate (Corning, United States). They were cultured in complete DMEM at 37°C with 5% CO_2_ for 3 days to generate tumor spheroids. After washed twice with PBS, 5 to10 tumor spheroids with a diameter around 500 μm were transferred into each well of a Falcon chambered cell culture slide (Corning, United States). An aliquot of either free Cy5-aptamer or Cy5-aptamer pre-incubated with 5×10^5^ CFSE-labeled (Invitrogen, United States) PBMCs was added into each well. After coincubation at 37°C for 4 h, unbound aptamer was washed out and cells were left in the culture medium for another 4 h. Subsequently, cells/spheroids on the slide were washed twice with PBS followed by fixing with 4% paraformaldehyde. Finally, tumor spheroids were imaged under a Fluo View™ 3,000 confocal microscope.

### Statistical Analysis

Statistical analysis was performed with the GraphPad Prism 8 software. Data is presented as mean ± s. d. Two-tailed, unpaired Student’s *t*-test was applied to compare values between 2 groups, and one-way ANOVA with Turkey’s correction was used to analyze results from multiple groups. For correlation analysis, data were fitted with linear regression, and Pearson correlation coefficients were calculated. *p < 0.05; **p < 0.01; ***p < 0.001; ****p < 0.0001.

## Results

### Aptamer is Associated With MDSCs in Circulation

**FIGURE 1:**
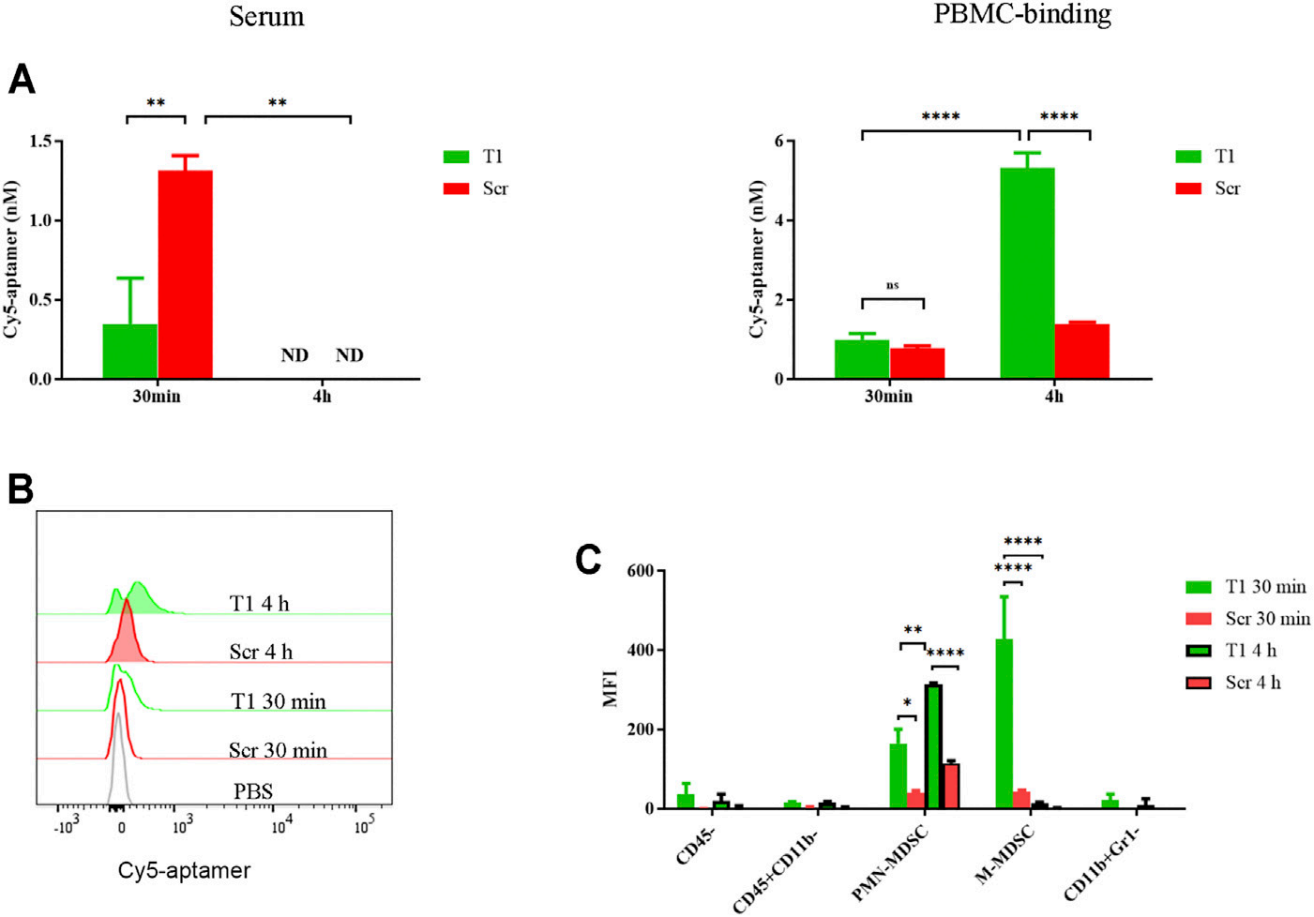
Partition of aptamer in circulation. 4T1 tumor-bearing mice were treated *i.v.* with 0.4 nmol Cy5-T1 thioaptamer (T1) or Cy5-scramble aptamer (Scr), and periphery blood samples were collected 30 min and 4 h later for fluorescent analysis. **(A)** Fluorescent intensity in serum and PBMCs. ND: not detectable. **(B)** Flow cytometry analysis of cell-associated Cy5-aptamers at the 30 min and 4 h time points. **(C)** Distribution of Cy5-aptamer among cell subsets in PBMC. MFI: median fluorescence intensity.

In a previous study, we detected binding of tumor cells and subsets of myeloid cells by the T1 aptamer (Liu et al., 2018). Here, we performed studies to further investigate aptamer-cell interaction *in vivo*. After mice bearing 4T1 tumors were treated with Cy5-labeled T1 or scramble aptamers, we detected dramatic decrease of T1 aptamer level in the serum within 30 min compared to the scramble aptamer control, and a surge of cell-associated T1 within 4 h (Figure 1A). Flow cytometry analysis confirmed cell-bound T1 aptamer (Figure 1B). Cell type analysis revealed that most T1 aptamers were associated with the CD45^+^CD11b^+^Ly6C^+^Ly6G^−^ monocytic MDSCs (M-MDSCs) and CD45^+^CD11b^+^ Ly6C^−^Ly6G^+^ polymorphonuclear MDSCs (PMN-MDSCs). In addition, there was an interesting shift of T1 aptamer-associated cells from M-MDSCs at 30 min to PMN-MDSCs at 4 h (Figure 1C). Given that tumor-bearing mice are overloaded with MDSCs in circulation, these cells provide the main source for retention of the aptamers. Since MDSCs tend to infiltrate into the tumor and support tumor growth, they may also serve as a vector for tumor-orientated transportation of T1 aptamers and hence T1-conjugated therapeutic agents (Ostrand-Rosenberg and Fenselau, 2018). Thus, it is necessary to further evaluate T1 aptamer and its binding activity with MDSCs (Hasegawa et al., 2016).

### Structure-Activity Relationship Analysis Reveals Key Structural Features of Aptamer

**FIGURE 2:**
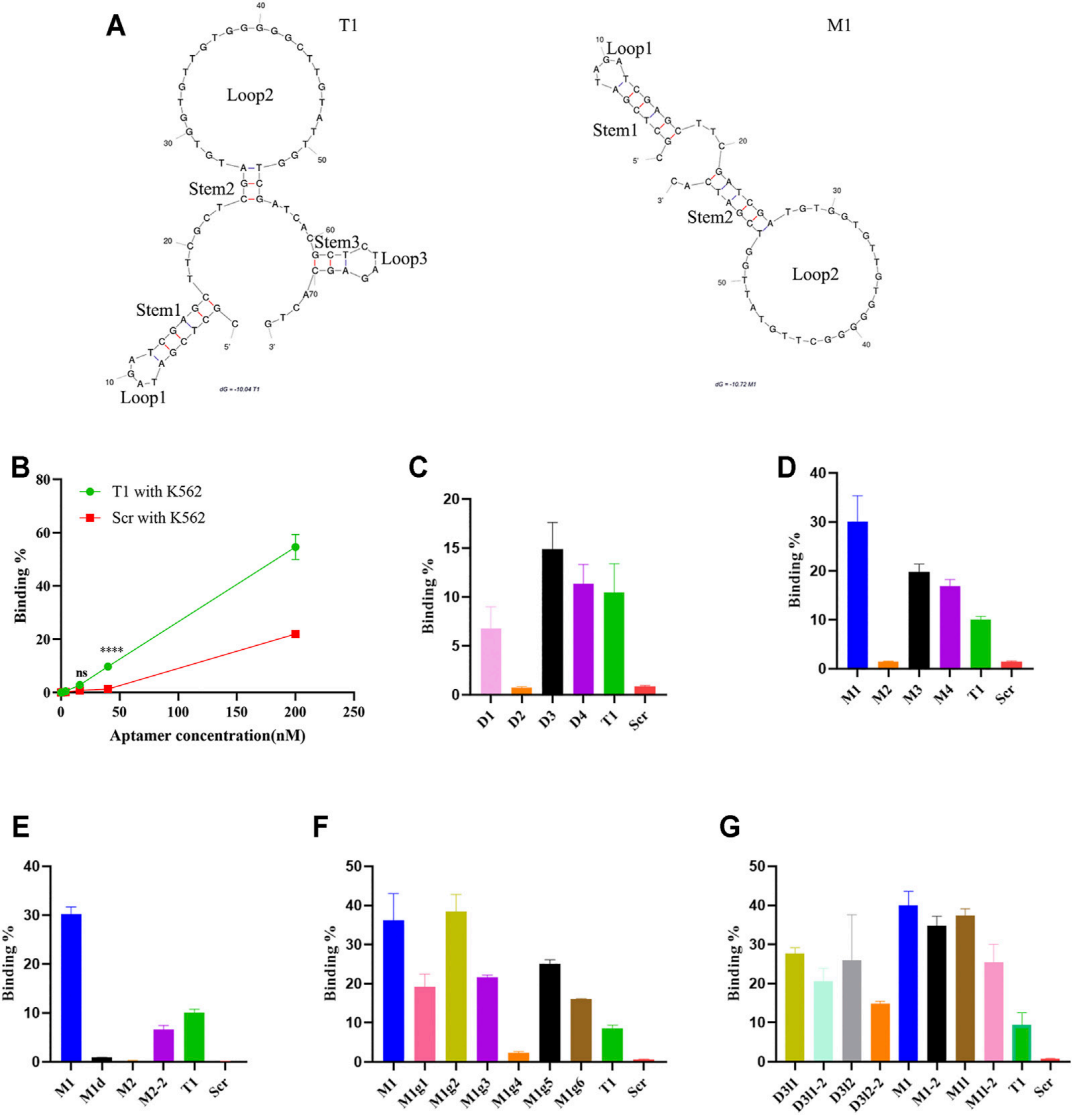
Analysis of aptamer binding to K562 cells. **(A)** Predicted secondary structures of T1 and M1 aptamers. Stems and loops in the aptamer are labeled. **(B)** Dose-dependent binding of aptamers to K562 cells based on flow cytometry analysis. **(C–G)** Flow cytometry analysis on binding of K562 cells by T1 and derived aptamers.

It is generally accepted that binding from an aptamer is dependent on its spatial structural adaptability, and in most cases, only a small part of it is responsible for direct docking with the target (Ruscito and DeRosa, 2016; Azri et al., 2021). Structure-activity relationship analysis has been applied to determine key binding site(s) in an aptamer (De Fenza et al., 2020). We applied a similar approach to map the cell-binding sites in the T1 aptamer. The probable secondary structures were determined with the Mfold software that is based on folding Gibbs free energy calculation. Based on the prediction, T1 aptamer primarily consists of three stem-loop hairpin motifs (Figure 2A). Subsequently, a series of modifications were made to narrow down the pivotal segments of the T1 sequence (Table 1, Supplementary Figure S1).

Cell-binding capacity from T1-derived new aptamers was measured using two human leukemic cells as surrogates. K562 is a myelogenous leukemia line, and Molm14 is a monocytic leukemia line. These cell lines bear close similarity with MDSCs since they all represent poorly differentiated myeloid cells. Overall, K562 cells displayed higher binding capacity to the T1 aptamer than Molm14 cells (Figure 2B, Supplementary Figure S2A). In the first set of study, we generated a group of new aptamers by truncating big pieces in the T1 aptamer. The D1 and D2 aptamers carried large deletions in the 5′ region (loop1 and stem 1) and the middle region (loop 2 and stem 2), respectively. D3 missed loop 3 and stem 3, and D4 had a larger sequence deletion than D3 (Table 1, Supplementary Figure S1). Truncation of the 5′ stem and loop (D1) resulted in partial loss of activity, while depletion of loop 2 (D2) caused a total loss of cell-binding ability. Interestingly, D3 and D4 retained cell-binding activity (Figure 2C, Supplementary Figure S2B), indicating that the 3′ loop 3 and stem 3 are not involved in aptamer-cell interaction.

D3 aptamer was selected for further modifications. It has been previously shown that the size of loop and stem has an impact on the function and stability of a nucleic acid hairpin (Vallone et al., 1999; Kuznetsov et al., 2008). Compared to the parental D3, M1 has an elongated stem 2 with six base pairs, while M2 has a dGGGGG deletion in loop 2 resulting in a smaller loop. With similar alterations in the 5′ region, M3 has a shorter stem 1, and M4 adopts an enlarged loop 1 (Table1, Supplementary Figure S1). Among these four new aptamers, M1 showed the highest binding capacity to the human leukemia cells, and M3 and M4 retained their cell-binding capabilities. Surprisingly, M2 completely lost its cell-binding activity (Figure 2D, Supplementary Figure S2C).

Based on the observations that depletion of loop 2 (D2) and deletion of the dGGGGG segment in loop 2 (M2) caused complete lose of cell-binding activity, we hypothesized that either the size of loop 2 or a specific sequence feature in the loop was essential for aptamer activity. Indeed, deletion of the dGGGGG segment in M1 (M1d) caused a complete loss of activity, while deletion of five nucleotides outside of the dGGGGG segment in loop 2 (M2-2) retained partial activity (Figure 2E, Supplementary Figure 2D). These results point to a pivotal role of the dGGGGG segment in cell-binding activity.

To further evaluate contribution from dGGGGG segment on cell binding, we mutated a number of nucleotides in loop 2 of the M1 aptamer to generate aptamers with additional G-rich segments or segments with different length (Table 1, Supplementary Figure S1). M1g1 contains two G-rich fragments while M1g2 has four of them. As expected, the two new aptamers retained high cell-binding activity (Figure 2F, Supplementary Figure S2). Reducing the length of the G-rich segment from five to three guanine nucleotides (M1g3) deprived M1 of its binding affinity, while extending the segment to 7 guanine nucleotides (M1g4) did not further enhance cell binding (Figure 2F, Supplementary Figure S2). In addition, transposition of the G-rich segment in loop 2 (M1g5 and M1g6) did not improve cell-binding activity either (Figure 2F, Supplementary Figure S2). These results strongly support the notion that a G-rich segment with a certain length in loop 2 is strictly required for aptamer activity. In the meanwhile, increasing the number of G-rich segments did not further improve cell-binding activity from M1.

Addition analysis was performed in aptamers with an undisrupted loop 2. The D3 derivatives (D3L1, D3L1-2, D3L2, D3L2-2) had elongated stem 2 over D3, and the M1 derivatives (M1-2, M1L, M1L-2) had extended stem 2 compared to M1 (Supplementary Figure S1). These derivatives had either comparable or inferior activities compared to M1 in a cell-based assay (Figure 2G, Supplementary Figure S2).

### Tetramolecular G-Quadruplex is Essential for MDSC Binding

**FIGURE 3:**
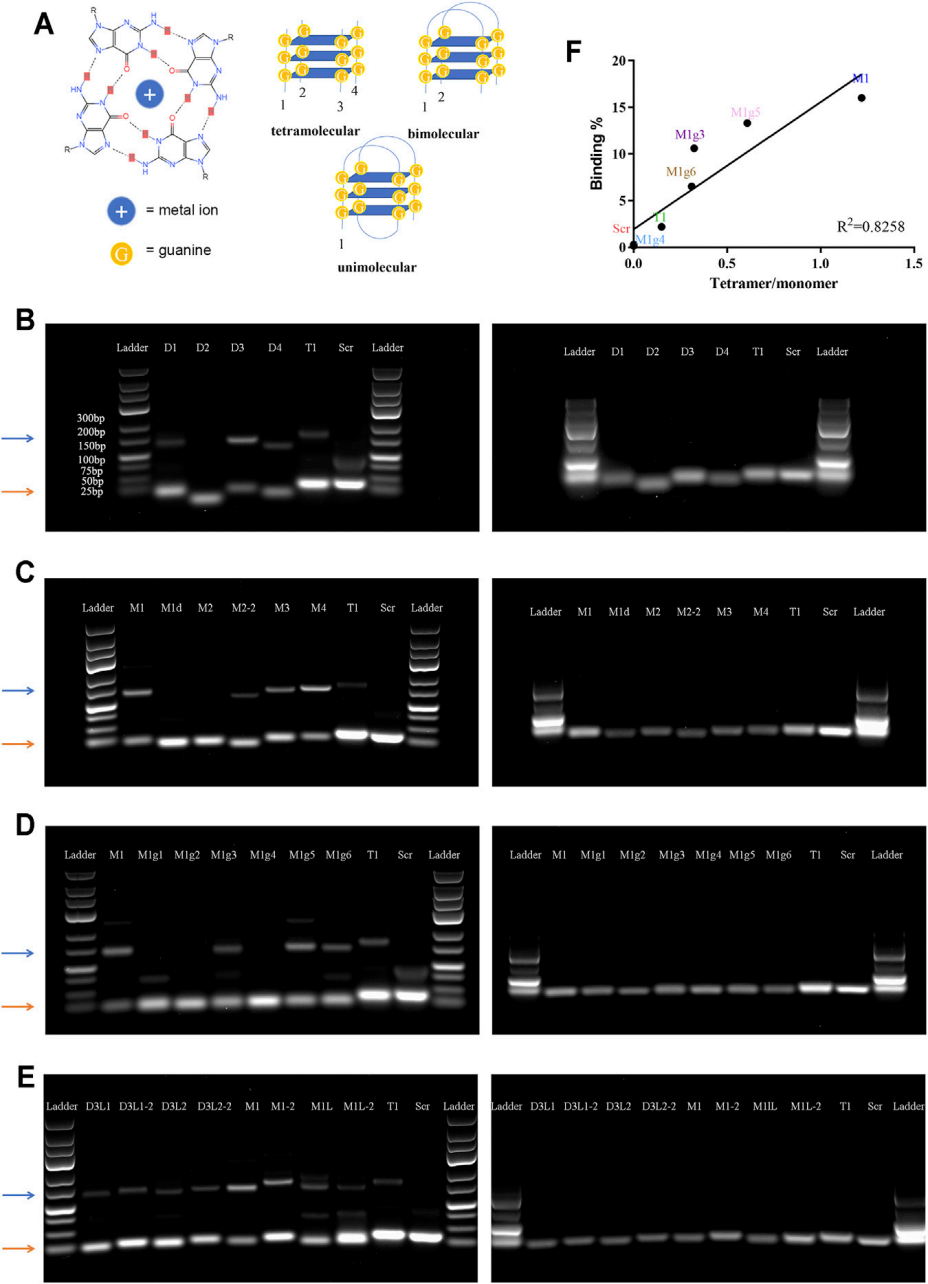
Analysis of tetramolecular G-quadruplex. **(A)** Schematic view of special secondary structures of G-quadruplex. Left panel: molecular structure of a G-quartets. Right panels: Secondary structures of unimolecular, bimolecular, and tetramolecular G-quadruplexes. **(B–E)** Images of agarose gel electrophoresis results. Left panels: non-denatured gels; right panels: denatured gels. Orange arrows point to the monomer bands, and blue arrows point to the tetramer bands. **(F)** Correlation between cell binding affinity and tetramer/monomer ratio.

It has been reported that G-rich oligonucleotides have the propensity to form a G-quadruplex (G4) structure under appropriate conditions (Kwok and Merrick, 2017). G4 is a non-canonical nucleic acid structure formed by stacking interaction of G-quartets where four guanines are assembled into a planar arrangement through hoogsteen hydrogen bonding (Figure 3A). Since aptamers with a G4 structure are more resistant to nucleases, G4 structures have often been incorporated into aptamer design (Bochman et al., 2012; Roxo et al., 2019). We applied web-based G4 Hunter service for G-quadruplex prediction to analyze the guanine-rich aptamers. This program has been successfully used to identify genome-wide G-quadruplex motifs and to correlate with their specific functions (Gazanion et al., 2020; Bohálová et al., 2021). The system assigned G4 Hunter score representing a quadruplex propensity in each sequence and predicted the number of putative quadruplexes (Table 1).

Since there is only one consecutive G-rich region in T1 and T1-derived aptamers, an intermolecular interaction is needed to form a G4 structure (Pedroso et al., 2007). To test this hypothesis, we performed agarose gel electrophoresis under both denatured and non-denatured conditions. Each aptamer showed one band on the denatured gel that correlated with the proper molecular weight; however, many aptamers had two bands on the non-denatured gel, one correlating with the molecular weight and the other a higher molecular weight (Figure 3B–E). Careful analysis revealed that all aptamers that showed two intense bands on the non-denatured gel carried a dGGGGG segment, a result that precisely confirmed G4 Hunter prediction (Table 1). Among the G-rich segment-modified M1 derivates, there was a linear correlation between intensity of the quadruple bands (displayed by a tetramer band/monomer band ratio) and cell-binding activity, with M1 showing the highest tetramer ratio and the highest binding capacity (Figure 3F).

### The M1 Aptamer has a High Tumor Penetration Potential

**FIGURE 4:**
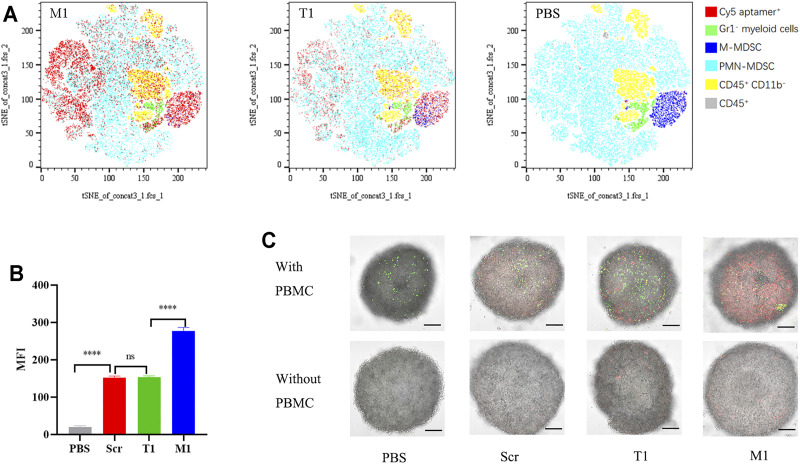
High MDSC-binding capacity from the M1 aptamer **(A)** tSNE map of T1 and M1 binding to PBMCs *ex vivo*. **(B)** Quantitative analysis of PBMC binding by aptamers. MFI: median fluorescence intensity. **(C)** Images of 4T1 spheroids with cell-transported aptamers. Upper panels: spheroids co-cultured with PBMCs pre-incubated with Cy5-aptamer. Bottom panels: spheroids co-cultured with free Cy5-aptamer. Green dots: CFSE-labeled PBMCs; red dots: Cy5-aptamer. Scale bar: 100 mm.

We performed an *ex vivo* assay to compare cell-binding capacity between the T1 and M1 aptamers. Both aptamers were applied to incubate with PBMCs derived from 4T1 tumor-bearing mice, and flow cytometry was performed to determine percentage of cells associated with the aptamer (Supplementary Figure S3). Interestingly, both aptamers were associated with the same pool of CD45^+^CD11b^+^myeloid cells (Figure 4A). However, binding capacity from M1 was twice as high as that of T1 based on fluorescent intensity from the cell-bound aptamers (Figure 4B).

To explore the feasibility of MDSC-mediated drug delivery, we performed an *in vitro* co-culture assay with PBMCs and 4T1 tumor spheroids (Supplementary Figure S3B). There have been many studies demonstrating the utility of tumor spheroids on interaction between tumor tissue and therapeutic reagents (Ibarra et al., 2020; Zheng et al., 2020). Confocal microscopic analysis revealed that CFSE-labeled PBMCs (in green) were able to penetrate deep into the tumor spheroids, with a concurrent increase of fluorescence from the Cy5-labeled aptamers (in red) hitchhiking inside the spheroids (Figure 4C). More importantly, fluorescent intensity was much stronger in samples treated with M1 aptamer than those with T1 or the scramble aptamer (Figure 4C), indicating that M1 was more effectively transported into the tumor spheroids by MDSCs.

## Discussion

In the current study, we performed structure-activity relationship studies to understand sequence requirement for our previously identified T1 aptamer on its binding to the poorly differentiated MDSCs. In the process, we identify new aptamers with improved binding capacity over T1, and M1 showed the highest MDSC-binding potential. Another interesting finding is that both T1 and M1 aptamers can bind to the circulating MDSCs. Since such cells are constantly recruited into the malignant tissue in support for tumor growth and metastasis, they can also serve as an ideal vehicle for intratumor drug delivery. Hence, both T1 and M1 can serve as precious reagents for tumor-targeted drug conjugates, and are expected to play important roles in cell-mediated tumor delivery of therapeutic agents. Based on our current study, M1 is more effective than T1 for the role.

An interesting feature of this set of aptamers is their ability to form tetramolecular G-quadruplexes. Our structure-activity relationship analysis confirmed the importance of the dGGGGG segment in forming a tetramolecular structure. Since the length and positions of loops and flanking sequences, together with other structural elements can all impact the stability of the tetramolecular structure, application of the G4 Hunter program provided systematic analysis for the T1-derived aptamers. In the meantime, we established a correlation between the special polymeric structure and its MDSC-binding capacity from the aptamer in the study. It is very interesting to observe a positive correlation between tetramer-to-monomer ratio and cell-binding activity (Figure 3F). It is highly likely that a unique tertiary structure containing the G-quadruplex is required for MDSC binding. Future study should be focused on confirmation of the tertiary structures and identification of the protein or protein cluster on cell surface that interacts with the aptamer. A recently reported fluorescence melting competition assay can be a useful tool in the study (Luo et al., 2021).

In conclusion, we have identified a group of aptamers with a high binding capacity to MDSCs. Among them, the M1 aptamer has the highest cell-binding capacity. This aptamer is expected to serve as a unique reagent for cell-mediated tumor delivery of therapeutic agents.

## Data Availability

The original contributions presented in the study are included in the article/Supplementary Material, further inquiries can be directed to the corresponding author.
