# Supplementary material for: Identification of an Aptamer With Binding Specificity to Tumor-Homing Myeloid-Derived Suppressor Cells
Source: Front Pharmacol. 2022 Jan 21;12:752934. doi: 10.3389/fphar.2021.752934 (PMC8814529; doi:10.3389/fphar.2021.752934)
Supplement: Supplementary file 1 [file DataSheet1.docx]

Supplementary Figure 1


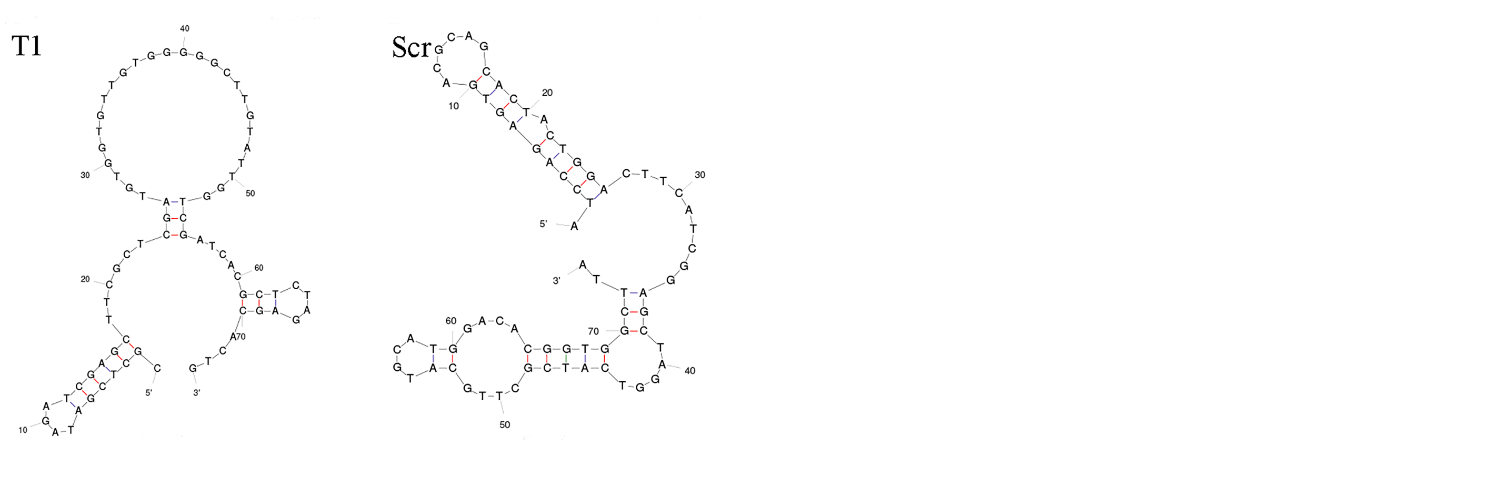


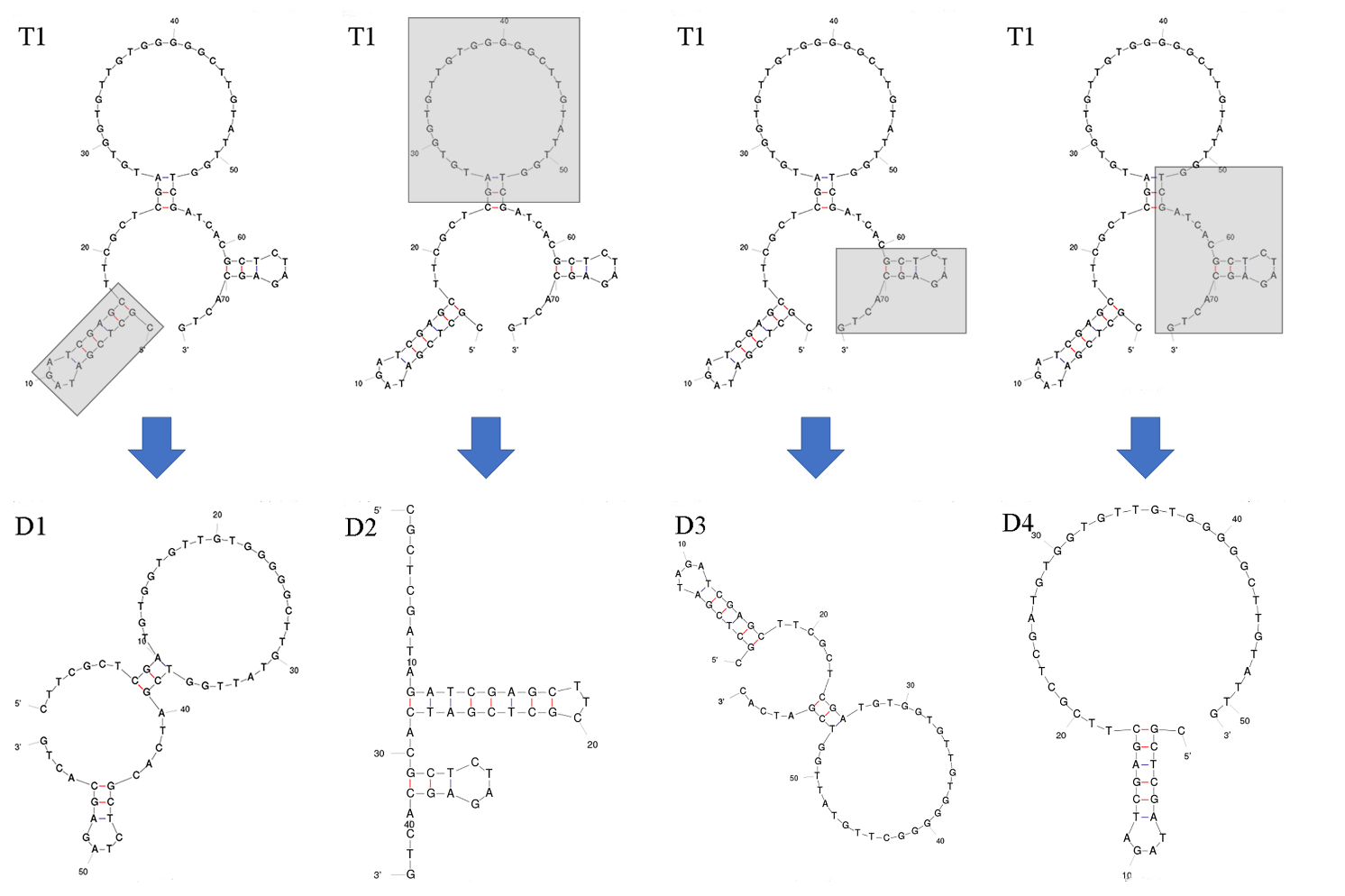


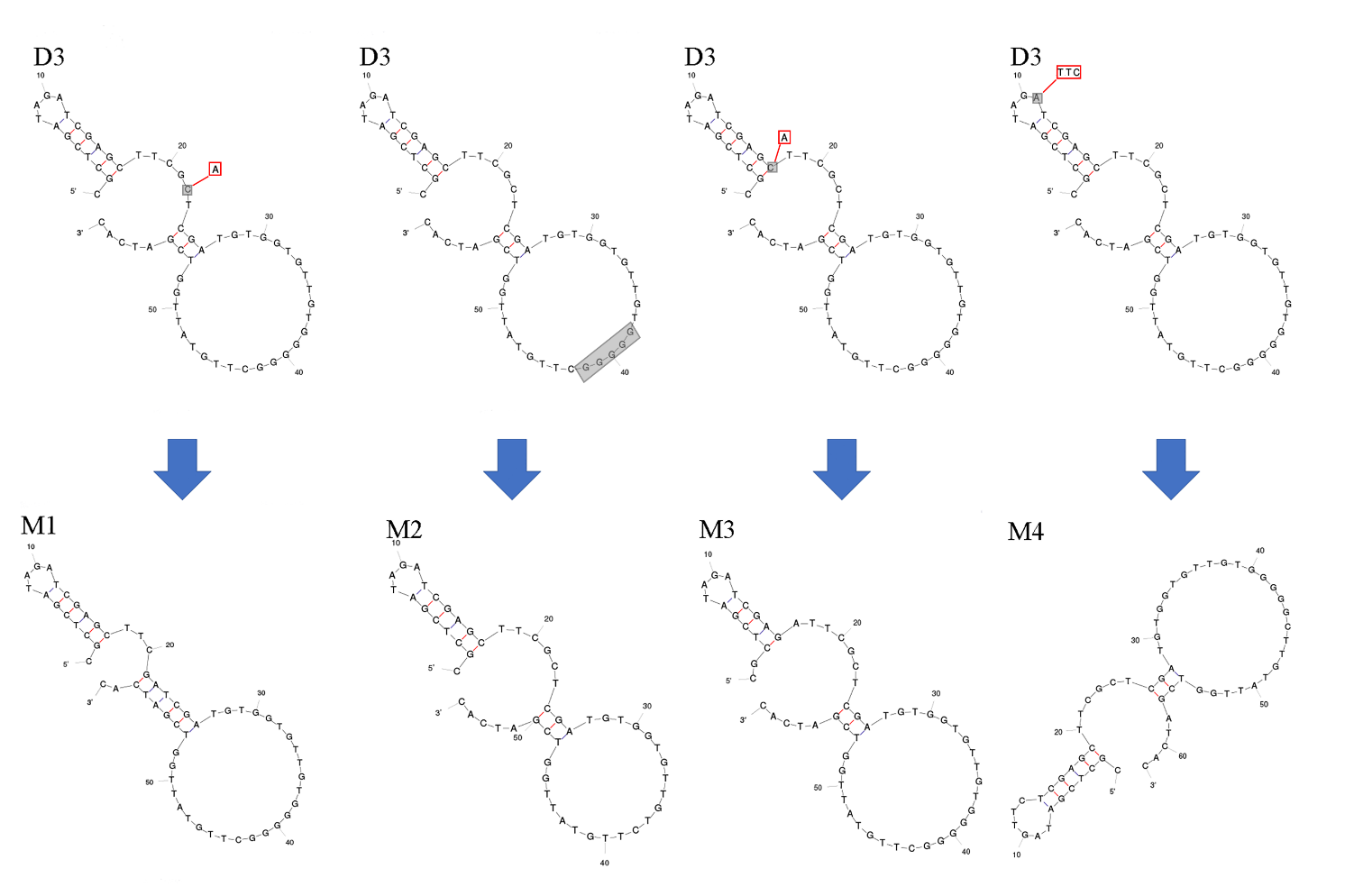


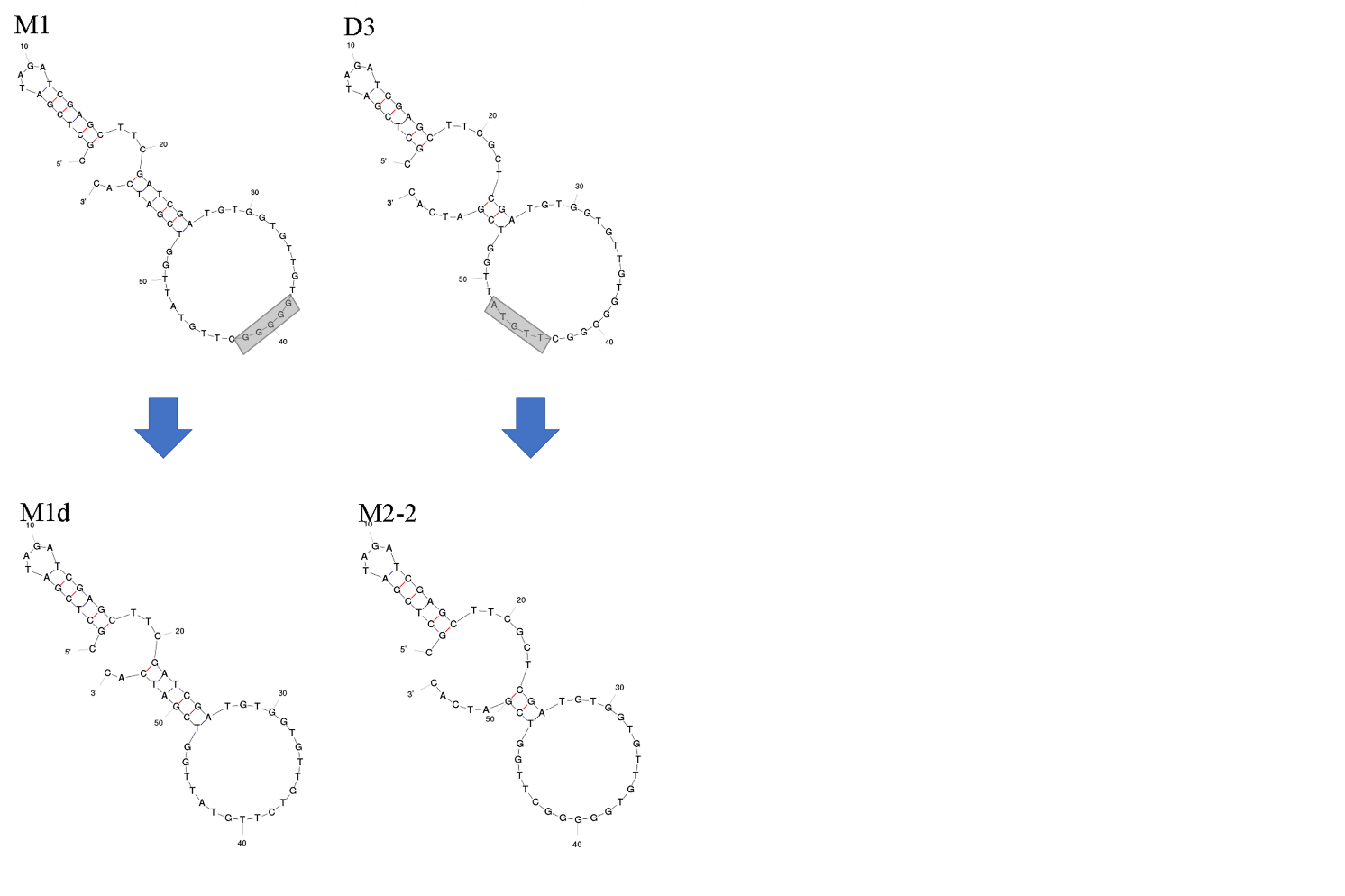


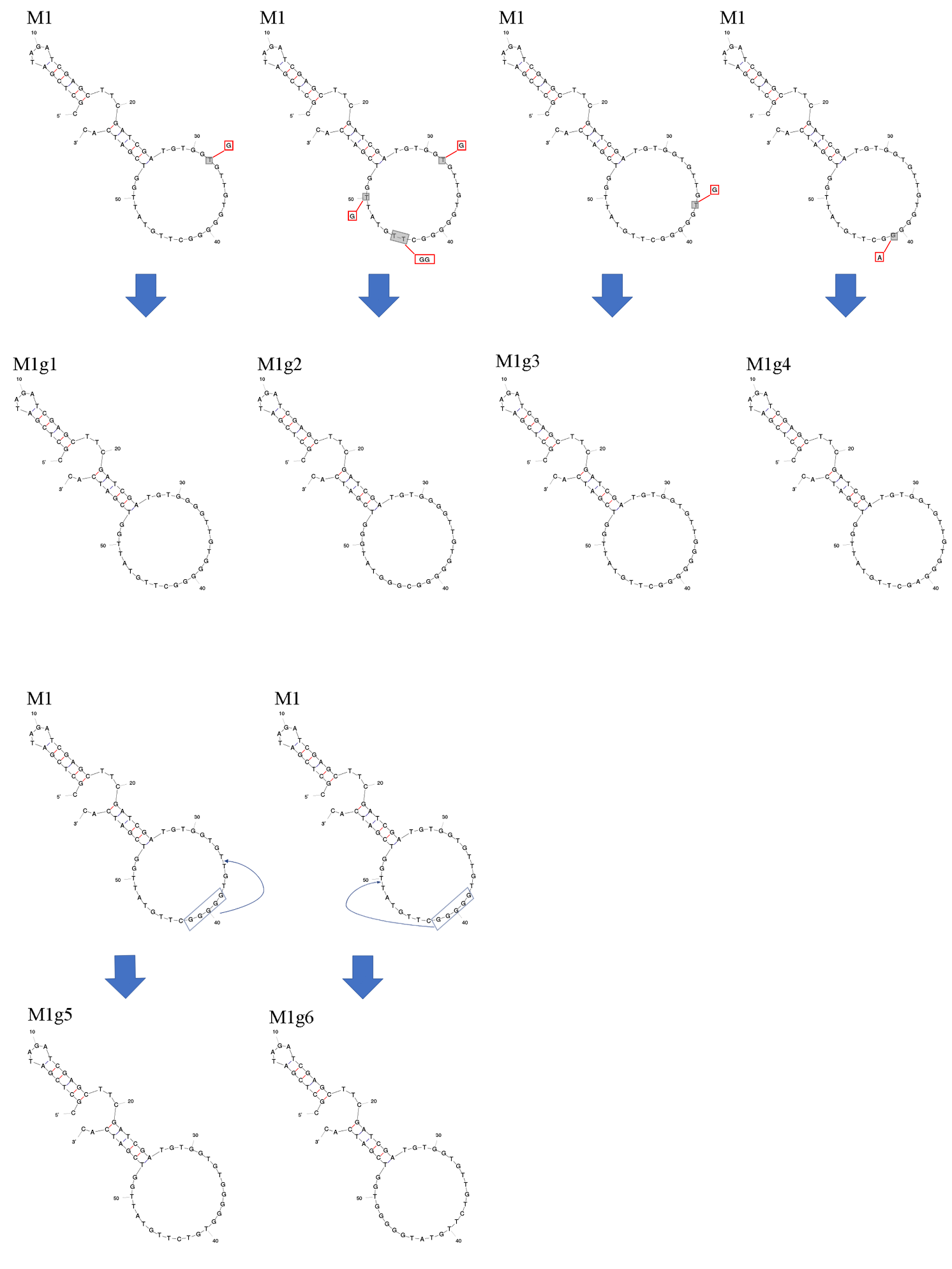


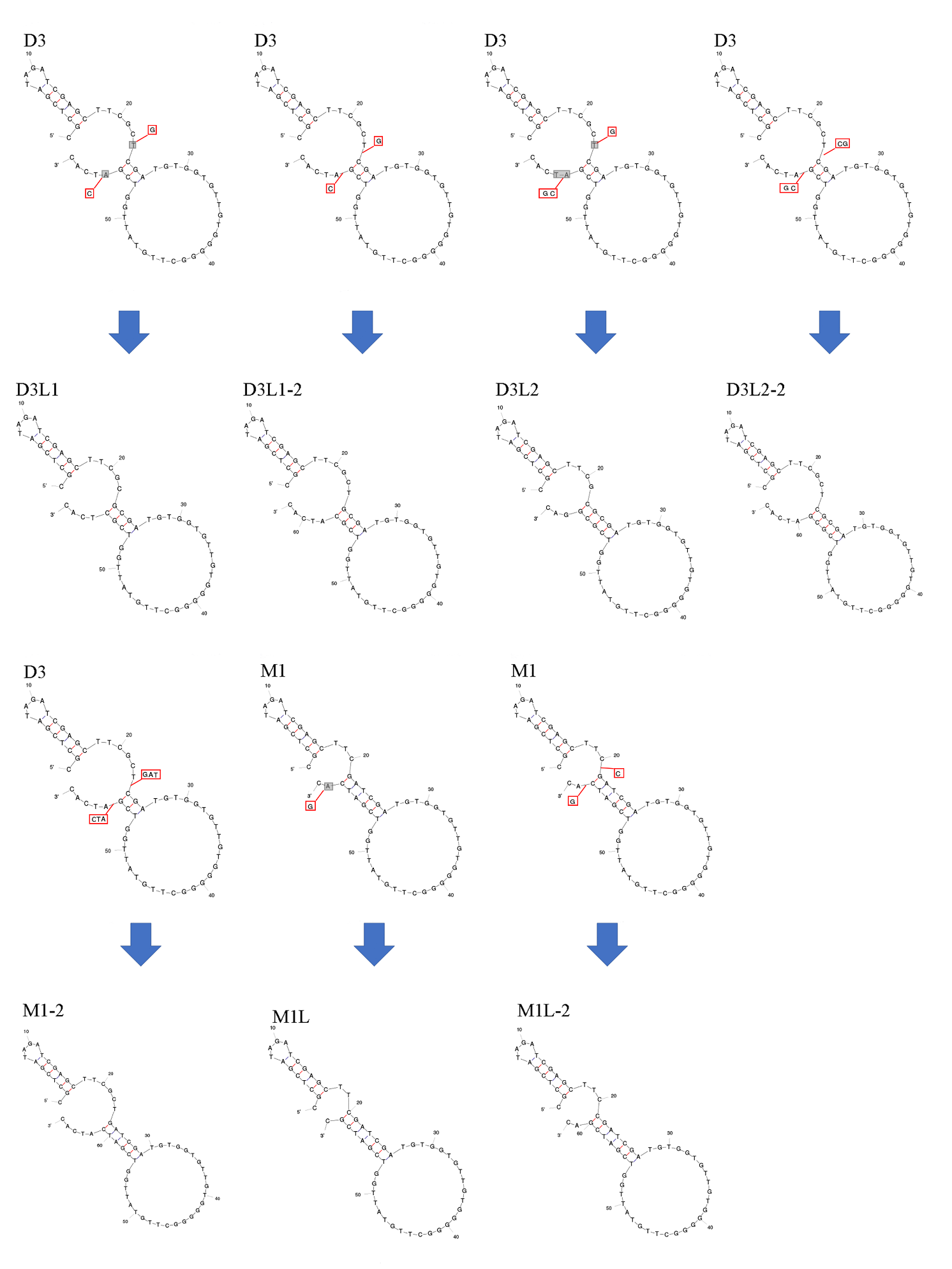


**Supplementary Figure 1. T1 aptamer, its derivatives and their predicted secondary structures.** Bold blue arrows point to the resulting new structures after sequence modification. Grey: The sequence set was removed; Red: The sequence set was inserted; Blue arrows: The sequence set was transferred to the indicated location.

Supplementary Figure 2


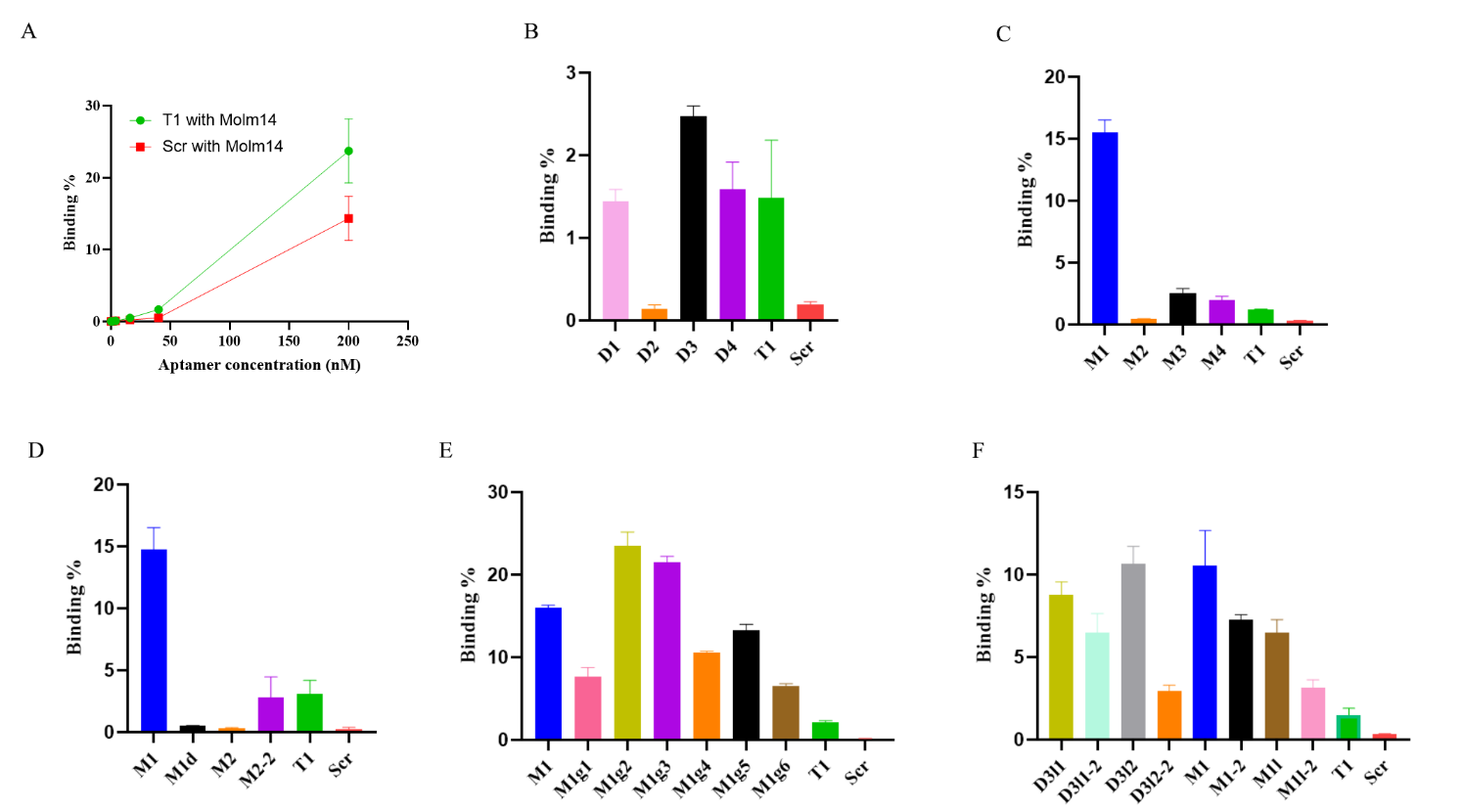


**Supplementary Figure 2. binding of Molm14 cells by aptamers.** (A) Dose-dependent binding of aptamers to Molm14 cells based on flow cytometry analysis. (B~F) Flow cytometry analysis on binding of Molm14 cells by T1 and derived aptamers.

Supplementary Figure 3


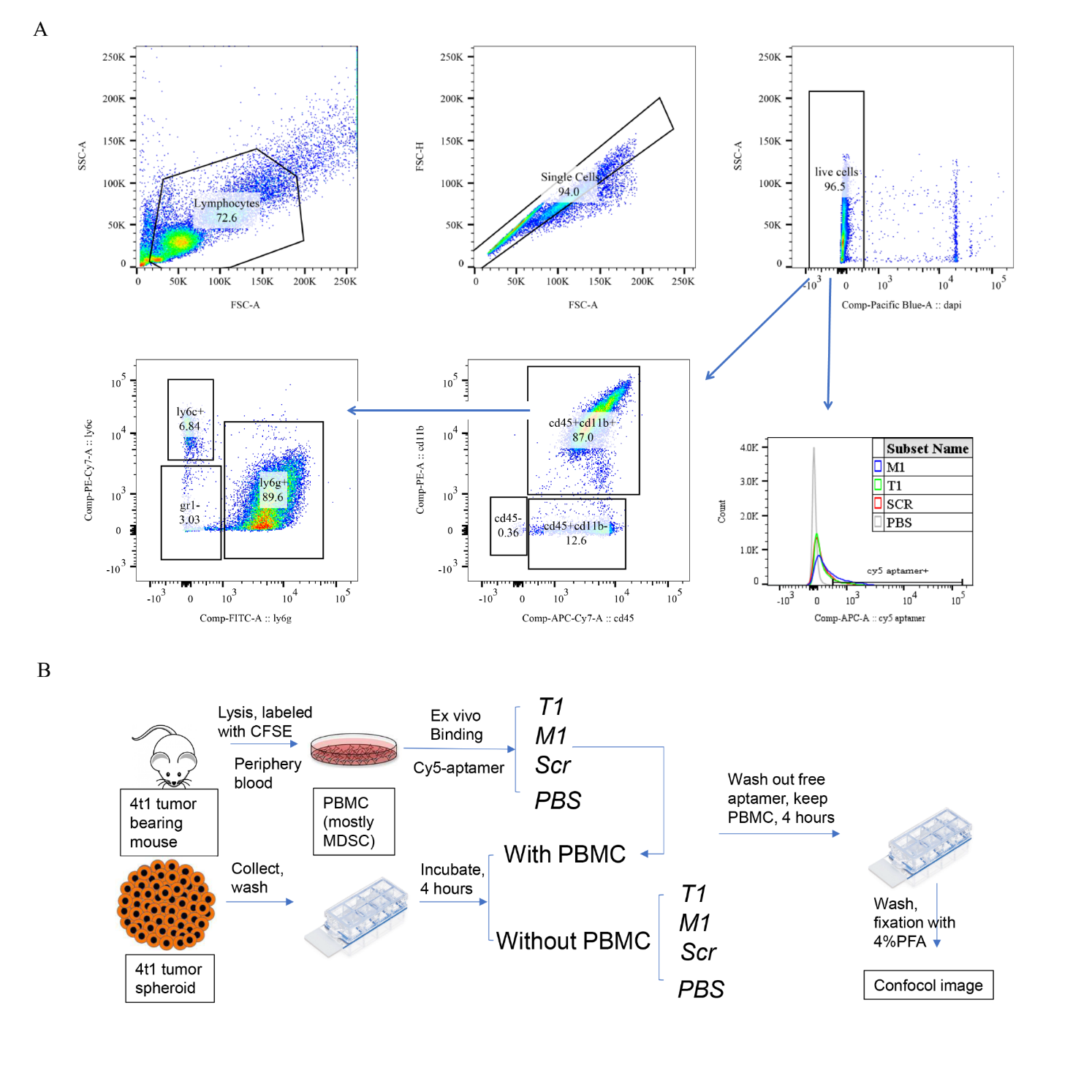


**Supplementary Figure 3.** (A) Gating strategy for flow cytometry analysis. (B) Schematic view of 4T1 spheroid penetration assay.
